# The Role and Limitations of the Reference Interval Within Clinical Chemistry and Its Reliability for Disease Detection

**DOI:** 10.3389/bjbs.2024.12339

**Published:** 2024-02-28

**Authors:** Nathan E. Timbrell

**Affiliations:** Nutristasis Unit, Synnovis, Guy’s and St. Thomas’ Hospital NHS Trust, London, United Kingdom

**Keywords:** clinical chemistry, reference intervals, reference ranges, normal range, clinical decision curve

## Abstract

Reference intervals (RIs) are a range of values that are supplied alongside laboratory measurements for comparison to allow interpretation of this data. Historically, RIs were referred to as the normal range. However, the perception of what is normal can lead to confusion in clinicians and unnecessary emotional distress in patients. RIs can be acquired using several methods. Laboratories may quote published studies or derive their own using established direct or indirect methods. Alternatively, laboratories may verify RIs provided by assay manufacturers using in-house studies. RIs have several limitations that clinicians should be aware of. The statistical methodology associated with establishment of RIs means that approximately 5% of “disease free” individuals will fall outside the RI. Additionally, the higher the number of tests requested, the higher the probability that one will be abnormal, and repeat results in an individual may show regression to the mean. Completion of studies for establishment of RIs can be expensive, difficult, and time consuming. Method bias and differences in populations can greatly influence RIs and prevent them from being transferable between some laboratories. Differences in individual characteristics such as age, ethnicity, and sex can result in large variation in some analytes. Some patients, such as those whose gender differs from that which was presumed for them at birth, may require their own RIs. Alternatively, a decision will need to be made about which to use. Overall, the issue common to these factors lies within interpretation. As such, RIs can be improved with better training in their use, combined with a better understanding of influences that affect them, and more transparent communication from laboratories in how RIs were derived.

## Normal Range or Reference Interval?

Reference intervals (RIs) are a range of values, usually derived from a population of “healthy” or disease-free individuals, to which results can be compared to aid interpretation. Essentially, it allows one to transform a number derived from an analytical measurement into clinically meaningful information. The RI has evolved over the years due to identification of limitations and introduction of new quality indicators and standards such as ISO1519:2012.

Before the 1960 s, laboratories worked in isolation meaning that they developed their own “normal” ranges. These normal ranges were derived from a poorly defined group of patients that were labelled as “normal.” The ranges were only applicable to their own patient populations and their methods to account for methodological variation [1]. The major limitation associated with these ranges was the use of the word “normal,” which can lead to incorrect inferences. Normal can be perceived to mean healthy or non-diseased. Alternatively, it could be referring to the distribution of results within a RI if they follow a normal or gaussian distribution [2]. It may also mean the most encountered, that which is often aspired to, or harmless [3]. From a patient perspective, it is not clear what “normal” is referring to, and to be told that one falls outside of a “normal” range could cause unnecessary emotional distress. Additionally, normality is relative and situational and, as such, “normal” ranges may not be applicable in certain patient groups [4].

Another major issue with the above method of RI establishment was the individuality of these RIs [1]. For the patient, this could lead to non-comparable results between laboratories if their care occurred in different regions. In a time where analyser diversity was limited, it would have made more sense if laboratories used common reference ranges published in textbooks or journal articles [5]. It is possible that a lack of inter-laboratory communication or method/procedural standardisation influenced the variability in so called “normal” ranges.

The concept of the “normal” range changed when Gräsbeck and Saris introduced the concept of a reference interval, also known as a reference range [6]. The pair attempted to improve laboratory ranges and shared their findings at a congress in 1969. In short, they thought that RIs should be derived from proper control subjects. The assumption being that the middle 95% of a set of results from a correctly defined “healthy” population would act as a RI to which laboratory measurements could be compared. Based on this, expert panels produced recommendations that aimed to improve the principles and terminology associated with RIs, while also improving overall laboratory quality and standardising RI determination. This led to the RI as it is known today.

## What is the Reference Interval and How is it Usually Established?

RIs are a selection of a set of results, usually the middle 95%, measured on a well-defined reference population [1]. The International Federation of Clinical Chemistry and Laboratory Medicine (IFCC) defines a reference population as a group made up of “reference individuals,” which are individuals that have been selected based on a set of criteria such as medical history, physical examinations, or laboratory investigations [7]. These definitions have also been approved by the World Health Organisation (WHO), Clinical & Laboratory Standards Institute (CLSI), and International Council for Standardization in Hematology (ICSH). A reference population is selected because it is not possible to measure an entire population. To navigate this issue, RIs can be derived using several methods.

**FIGURE 1:**
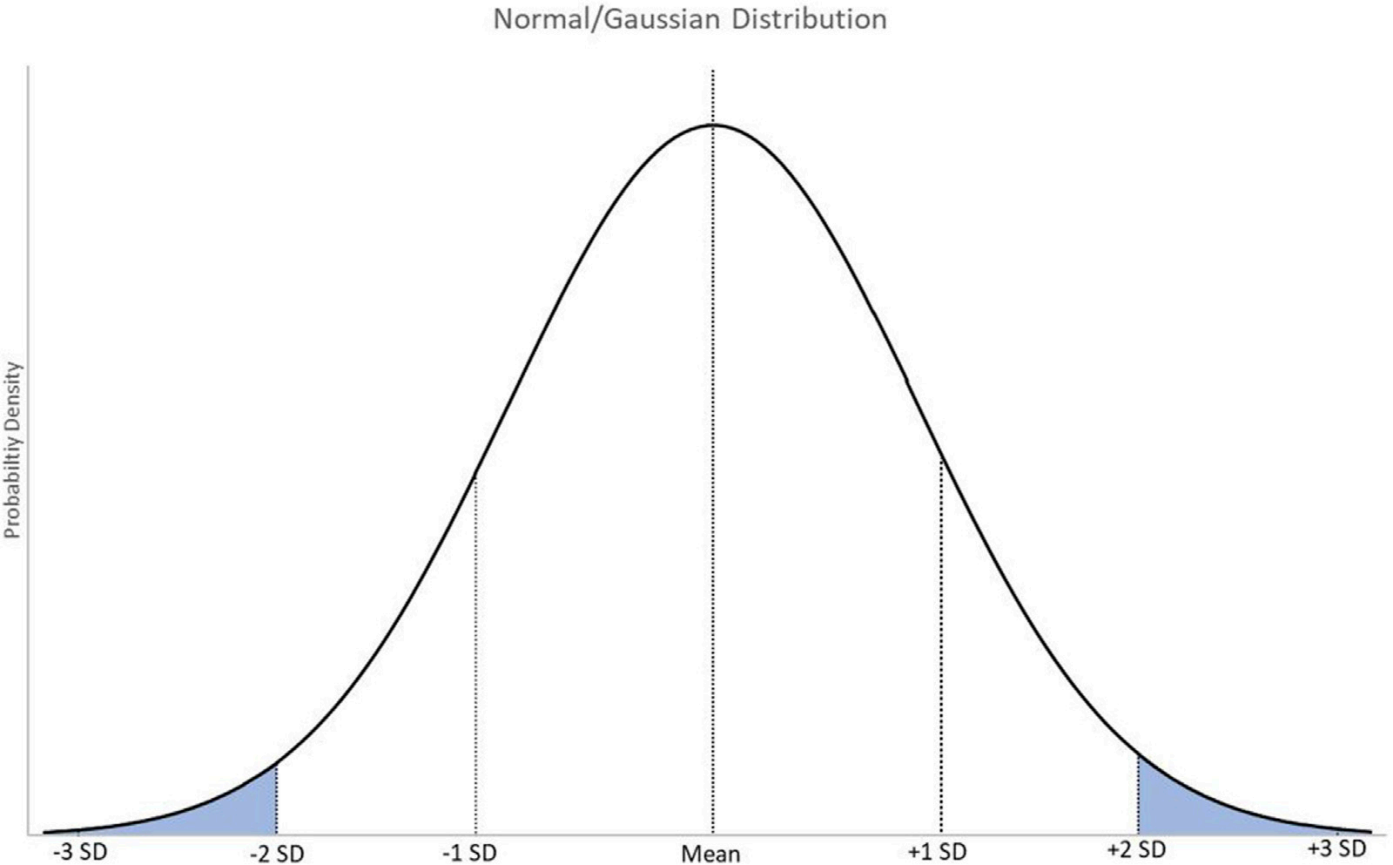
A graphical representation of normally distributed data with markers at the mean, and 1, 2, and 3 standard deviations from the mean. SD = standard deviation.

The simplest RI assumes data will follow a normal or Gaussian distribution where results are spread equally around a central mean (Figure 1) [8, 9]. Outliers are removed and the central 95% is determined as the RI. 95% is equal to approximately 2 standard deviations from the central mean. However, this means that 5% of this disease-free population falls outside of the RI, with 2.5% above the upper reference limit (URL) and 2.5% below the lower reference limit (LRL). Confidence intervals, usually 90%, can also be supplied. This identifies to clinicians at a specified confidence level the range within which the “true” reference limit lies. Essentially, this is a measure of the error associated with the limits of the RI.

Not all data follows a normal or gaussian distribution [1]. Some data may be skewed or curved and require mathematical transformation. A successful transformation will convert the non-Gaussian distribution into a normal distribution meaning the RI can be determined as discussed above. If the data can’t be transformed successfully, non-parametric methods can be used to determine the middle 95% of individuals [2]. These methods make no statistical assumption about the data that is being analysed. At their simplest, non-parametric tests involve obtaining a selection, usually the 2.5th and 97.5th percentiles, of the dataset. The complexity of datasets can mean that computation procedures may be required to create distributions of the results to allow for calculation of these percentiles. The work of Kim *et al* is a useful example of this [10]. While estimating RIs for routine laboratory tests, they used both parametric and non-parametric methods. The parametric test involved transforming the data to make it normally distributed, obtaining the 2.5th and 97.5th percentiles, and then back transforming these into real values to act as the upper and lower limits. The non-parametric method involved distributing the data using a quantile-quantile graph and a histogram, excluding outliers, and then defining the 2.5th and 97.5th percentiles as the upper and lower limits. Overall, they found that most RIs were comparable for full blood count parameters, however the exclusion process in the non-parametric method led to lower upper limits for some clinical chemistry analytes. This highlights how the methods used to establish RIs can lead to differing results.

A criticism of these methods is that there is no underlying theory evidencing that the central 95% is the best interval to use and that it is just based on the idea that 2 standard deviations from the mean is suitably distant. One could argue that the central 99.9% of individuals could be used as it would mean that only 0.1% of the disease-free population would fall outside of the RI. In fact, Jørgensen *et al* argued that RIs should reflect a true negative and therefore the 99.9th percentile is a better choice than the 95th [11]. They claim that doing so would reduce the probability of a false result occurring in 10 sequential tests from 40% to 1%. However, the consequence of using such a high percentage to define a RI would then be much more dependent on how well established the RI is. For example, if 20 individuals have been used to establish this RI, the likelihood of a false negative would be high as they probably wouldn’t reflect the true population. Overall, the central 95% of a well-defined and large enough disease-free population is likely to give the most appropriate balance between true and false diagnoses.

The past 30–40 years have seen several key publications and guidelines related to RIs. The 3rd edition of CLSI RI guideline EP28-A3c, titled “Defining, Establishing, and Verifying Reference Intervals in the Clinical Laboratory,” was published in 2008 and is still in use today [12]. It describes a protocol for determining RIs in a way that “meets the minimum requirements for reliability and usefulness.” Within this document, the recommendation is that a minimum of 120 individuals form the sample for a reference population. This number was derived by the CLSI working group and is recommended to ensure that both the central 95% of values and the 90% confidence intervals (CIs) could be calculated with statistical significance. Establishing a RI with 120 individuals would mean that the central 95% would include 114 measurements, and both the 2.5th percentile and 97.5th percentile would include 3 measurements each. Using less than 120 measurements will mean the confidence intervals will be extremely wide. In fact, Haeckel *et al* suggest that the CLSI recommendation of 120 individuals is a limitation due to the number being too small leading to broad CIs [13]. However, it is important to remember the practicality of performing RI studies with large sample sizes.

Ideally, individuals selected as part of a reference population should represent the population that the laboratory is serving [12, 14]. Selecting a reference population of mostly white males and females aged 25–60 when the population served is composed of multiple ethnicities and a wider age range would be unsuitable. For example, people of Black ethnic origin are recognised to have higher vitamin B12 concentrations than other ethnicities yet RIs are mainly derived from white populations [15]. It is likely that this has historically led to underdiagnosis of B12 deficiency and a lack of identification of associated comorbidities. If necessary, groups can be partitioned to derive individual RIs. Within reference populations, selected individuals should act as a suitable “control” group [12, 14]. This means that they should be disease free, or at least should not have any disease states that may influence the analyte to be measured. While some diseases may have an obvious classification of testing positive or negative and therefore may be easy to identify, it is important to think about how overt or subclinical disease could affect the population being studied. For example, cardiac troponin is used to assess individuals with acute coronary syndrome. However, it is elevated in a range of other situations including, but not limited to, cardiac surgery, cardioversion, aortic dissection, sepsis, critical illness, pulmonary embolism, and extreme exertion [16]. If the desire were to establish a more practical RI for acute coronary syndrome, one would have to consider if these individuals should be included in the reference population.

For a RI to be useful to a laboratory, the pre-analytical and analytical conditions used during its determination should match those that are in use by the laboratory. The aim of this is to remove result variability that is not due to the standard variation in a population [12, 14]. There are pre-analytical factors, such as storage time and time until centrifugation, that can influence measured analyte concentration. For example, pseudohyperkalaemia, a spuriously elevated potassium, can be caused by factors such as haemolysis, temperature during transportation, fist clenching during sample taking, and traumatic venipuncture [17]. Similarly, analytical factors may also introduce variability. Progress has been made in laboratory medicine so that results are comparable when measured in different laboratories. However, technology available in clinical laboratories may vary and results generated could differ despite progress made in metrological traceability of standard material used in calibrators [18]. Therefore, it is important to consider the methodology used to establish a RI and if it is appropriate.

The aspects discussed above highlight that the design of a standardised procedure for RI determination is critical. Scientists must identify a suitable reference population and a suitable method of analysis that reflects the pre-analytical and analytical aspects of result generation, and they must select methods for data analysis that are appropriate to collected data. Also of consideration, the requirements of ISO15189 require that RIs are reviewed on a periodic basis, when a pre-examination or examination procedure is changed, or when an existing RI is deemed unsuitable [19]. Therefore, establishment of RIs is not a “one and done” process, it is an evolutionary process and requires regular review to ensure that those in use are still appropriate.

## The Use of the Reference Interval in Clinical Chemistry

Clinical chemistry is a discipline in which most reported results are quantitative, meaning they are a set of numbers. These numbers are used to describe the function of various organ systems (e.g., liver and kidney). Without RIs, many of these generated quantitative measurements would be difficult to interpret. For example, a result of 51 arbitrary units for C-reactive protein and ferritin would be useless without a RI as there would be nothing to compare it to. How would a clinician determine if further investigation is required with this information? By supplying RIs, laboratories enable the clinician to make informed clinical decisions. In the example given, C-reactive protein would be highly elevated whereas ferritin could be considered “normal” [20, 21]. One would most definitely require further investigation, and the other wouldn’t in most cases. Therefore, RIs are an extremely valuable reference. However, one could argue that the clinical picture is equally, if not more, important than the laboratory measurements on a patient.

The RI is used by laboratory staff and other healthcare professionals in several ways. Firstly, as discussed above, it can be used to interpret the clinical situation based on a measured result. Interpretation can be left solely to the individual viewing the result by using their own expertise, or interpretative comments can be attached to results in an electronic patient record or on a report to aid interpretation. In fact, RCPath have produced guidelines for the provision of interpretative comments [22]. Comments can be added manually by suitably trained laboratory staff using the RI and clinical details as a guide. For example, a urea and electrolyte panel may require discussion of likely causes of abnormalities, such as primary polydipsia related hyponatraemia [23]. More commonly due to the high volume of tests, automated addition of comments occurs via algorithms existing within the laboratory information management system (LIMS) and laboratory staff can amend as required on a case-by-case basis. These algorithms may be determined by established RIs.

Secondly, RIs can be used to identify issues related to measurement. Computer algorithms can be set up in LIMS that withhold results and prevent their release to requestors. A common rule is the “delta check” which is a limit on the difference between two measurements for the same analyte [24]. A change in result outside of these set parameters will cause a result failure and lead to it being held for review. Alternatively, results may be held for review if they fall outside of a RI by a set amount. This will allow laboratory staff to check if these results are spurious. For example, K^+^ ethylenediaminetetraacetic acid contamination can alter several biochemical parameters. If contamination is suspected, calcium or alkaline phosphatase measurements can be added on for confirmation [25]. Without RIs, this would be difficult to identify.

Thirdly, RIs can be used by LIMS to automatically add on additional tests based on algorithms, a process known as reflex testing. If results are outside of the limits of the RI, tests could be added on within the LIMS to help the requestor identify the cause. This would eliminate the time it would take for the result to be seen and either a request for an add-on test to be made or an additional sample processed. Examples of common reflex tests include thyroid function tests such as free T4 following a thyroid stimulating hormone result outside of the RI, macroprolactin following a prolactin result above the upper limit of the RI, and magnesium following a potassium or calcium measurement below the lower limit of the RI [26]. Clearly, RIs are critical to the clinical chemistry laboratory. However, they have several limitations that those involved in their use need to be aware of.

## Limitations of the Standard Reference Interval

The standard RI has limitations that can severely impact the interpretation of results and, therefore, the quality of care received by an individual. While the well-known claim that pathology investigations are involved in 70% of healthcare decisions is now disputed, it is almost certain that laboratory testing has a major role in healthcare decision making [27]. It is likely that most of these decisions could be based on interpretation of data using RIs. Therefore, an awareness of their limitations is crucial.

The standard RI suffers from several issues relating to statistical methodology. As discussed above, 5% of “disease free” individuals will have a measured result that falls outside of the RI leading to possible misclassification of their disease status, also known as a false positive or negative [1, 28]. In other words, the probability that a result on a “disease free” individual is outside of the RI is 1 in 20. Additionally, each analytical measurement has a level of uncertainty associated with it due to systematic and random error. Repeat measurements on the same sample will not be identical and the dispersion will follow a normal or gaussian distribution. Therefore, a measured value should be considered as an estimate of the true value [29]. The above disease misclassification rate is related to the statistical methods associated with RI estimation. When combined with the measurement uncertainty, the rate of false negatives or positives could be higher. This suggests that a significant proportion of individuals could be misdiagnosed if based solely on an individual test. Of course, most requests will be for panels of tests. Whyte and Kelly argue that the chance that one of these tests is abnormal can be assessed using a binomial distribution, a probability distribution that models situations with two outcomes [1]. In this case, the two possible outcomes are within or outside of the RI. A healthy patient has a 1 in 20, or 5% chance of a result falling outside of the RI. Assuming a binomial distribution, the probability that all tests will be within the RI in a panel can be calculated as follows:

$${\left(1-p\right)}^{n}$$

Where *p* is the probability of a result outside of the RI, and *n* is the number of tests in the panel.

**FIGURE 2:**
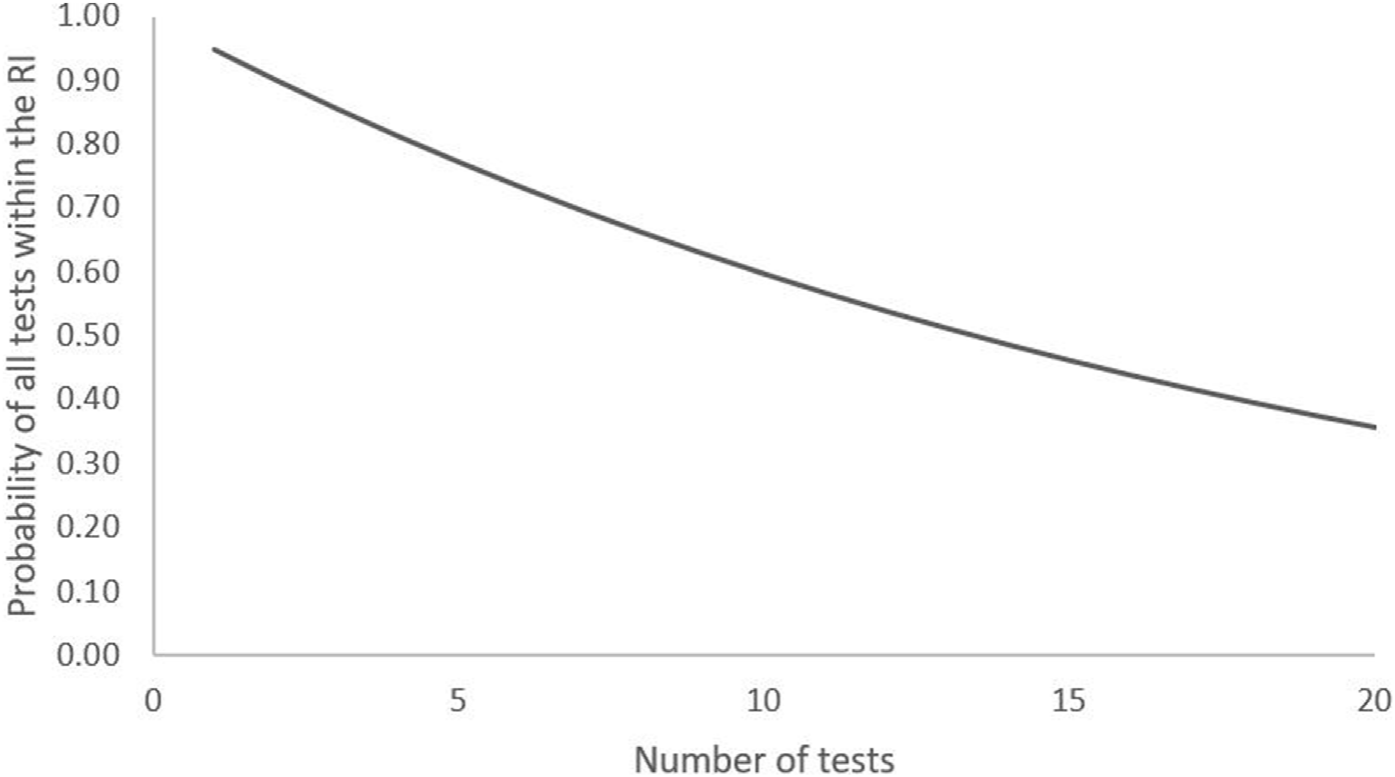
Probability of all results of a series of tests being within the RI in a disease-free individual.

Therefore, the likelihood of a test result falling outside of the RI increases with the number of tests in the panel, assuming they are independent (Figure 2). For example, the chance that 2 or 10 results on a disease-free individual are within the RI is 90% and 60% respectively. A limitation of Whyte and Kelly’s approach is the assumption that all tests are independent of each other. However, it does work as a thought-provoking example of the possible limitations of repeat testing using a statistically derived RI. In practice, it is logical that the more an individual is tested, the more likely a result outside of the RI will be measured.

Finally, regression to the mean is a statistical phenomenon that can occur during repeat testing. If a single result is unusually large or small, it tends to be followed by a repeat measurement that is closer to the mean [1, 30]. This causes a practical issue as it impacts the ability to distinguish a real change. In patients, this could lead to issues with monitoring efficacy of treatment. While this isn’t an issue specific to RIs, it can make result interpretation difficult if a result was originally outside the RI and a repeat measurement is within.

Another limitation is the difficulty associated with producing RIs. Guidance suggests that laboratories should establish their own RIs where possible due to differences in standard operating procedures [14]. As discussed above, the recommended number of measurements required to produce a RI is 120 “disease free” or “healthy” subjects [12, 14]. A laboratory looking to establish their own RIs for a method could have great difficulty acquiring 120 “healthy” subjects due to the effort and ethics required to organise the study. An alternative is to adopt a previously derived RI. However, there are some aspects to consider with this approach. What is the source of the RI? Is it derived from previously published literature? If so, are the method and patient population suitably similar? Perhaps the manufacturer supplied a RI based on their own RI study. If so, does the patient population match the one served by the clinical laboratory? There will be no RI supplied if it is a newly developed in-house method, so what should the laboratory staff do? The CLSI EP28-A3c guidelines provide recommendations for the verification and transference of a RI [12]. RIs being considered should be screened for suitability for use by performing a method comparison that involves analysing samples with results spanning the RI and measuring range. The agreement between methods is assessed using suitable statistical analyses, such as a linear regression with scatter and bias plots to assess variation at different areas of the measuring range [12, 31].

After successful screening, CLSI EP28-A3c guidelines recommend that the extent of the verification required is dependent on the pre-analytical processes, population served, and the method in use. If these aspects are very similar, further RI verification is not required and the RI can be implemented. If the pre-analytical processes, population served, and the method in use differ in the RI being considered, RIs must then be verified.

Three different approaches are recommended for verification [12]. If the methods are deemed close enough in performance, a subjective assessment could determine acceptability. Arguably, this is not a suitable approach in a world of rigorous accreditation standards, and it could be difficult to defend an implementation if the final decision was subjective and not sufficiently backed up by evidence and data. The second approach is to perform a small, in-house study involving analysing samples collected from 20 individuals from a suitably defined reference population. If partitioning is required, it is recommended that samples are collected from 20 individuals per partition. Outliers should be removed using a suitable statistical approach, and results compared to the RI. The RI should be accepted, re-tested, or rejected if ≤2, 3-4, or ≥5 of the measurements falls outside of the RI respectively [31]. Fewer samples than this could result in weak conclusions based on insufficient data. The third option suggested by the guidelines is to perform the same study but instead use a larger reference population (e.g., *n* = 20–120). Whilst the third approach involves assessing more data and could provide stronger evidence, it could still be difficult for laboratory staff to organise and may be unnecessary considering the second approach will also provide sufficient data.

An issue with establishing, screening, or verifying RIs is that there can be great difficulty in defining a “disease-free” or “healthy” state. Some diseases may have a pre-symptomatic period where disease onset has initiated but there is no clinical change visible. For example, prediabetes is a state of disturbed glycaemic control that is still below the threshold to diagnose diabetes. However, prediabetic individuals may be asymptomatic and only present with altered fasting plasma glucose and/or impaired glucose tolerance during an oral glucose tolerance test [32]. Interestingly, the World Health Organisation and the American Diabetes Association have different cut-off values for these tests [33, 34]. This highlights the difficulty in defining a “diseased” state even for major organisations. Additionally, there may not be sufficient information on the impact of other factors on the measurand. For example, a laboratory wishing to create a RI for folate may struggle to identify a true RI if their population is ingesting fortified food sources. These aspects can severely impact a laboratory’s ability to recruit appropriate subjects to establish their own RI.

Method bias is another limitation of the RI. If a laboratory can’t establish their own RIs it could be beneficial to use published data and verify it using the methods described above. Meta-analyses of normative data have been conducted with the aim to establish RIs in “healthy” populations using the pooled mean of multiple studies [35]. Some methods account for natural variation in a population both within and between studies [36–38]. Others use the confidence interval which reflects uncertainty in the mean rather than natural population variation [39]. However, a major limitation of using published data is that the analytical methods or population used to establish the ranges may differ greatly. This could especially be the case for methods developed in-house. Lee *et al.* examined the possibility of using a single harmonised RI [40]. Serum from healthy volunteers (*n*=>120) was collected and measured using 23 tests on the Abbott Architect c16000 analyser (spectrophotometric methods) and 13 tests on the Abbott Architect i2000SR analyser (immunoassay methods). Results were compared to manufacturer RIs and RIs developed by UK Pathology Harmony (UKPH). UKPH is a group that aims to establish common RIs for assays with the idea that minor method differences across laboratories are unlikely to have a significant clinical effect [41]. The calculated URLs and LRLs were found to differ from PH by >10% in 50% and 25% of c16000 and i2000SR tests respectively. When compared with manufacturer RIs, a >10% difference was observed in 40% of URLs and 36% of LRLs in c16000 tests, and 47% of URLs and 85% of LRLs in i2000SR tests. When the authors examined the RIs quoted by the manufacturer, they found that many of them were taken from published literature. Another study examined ferritin assays from 5 different manufacturers (Beckman Coulter, Roche, Ortho, Siemens, and Abbott) and found significant method bias even though traceable standards had been implemented in an attempt to standardise methods [42]. Comparison of Beckman and Roche methods showed differences in results ranging from 31% to 57% at clinical decision points. Comparison with an Ortho method showed that it underestimated results by −12% to −19% at decision points. These examples highlight the influence that method bias can have on RIs. From a physician standpoint, this will be particularly important in patients that are monitored with blood tests performed across laboratories at different sites with different methods, or if laboratories change their analysers or methods. In these cases, results from different methods may not be comparable. This, of course, can be very confusing for healthcare staff and could lead to over or under treatment. Considering the above, it is good practice for scientists to establish their own RIs for non-standardised methods. Additionally, scientists should provide advice to clinicians and service users to help them interpret results and understand differences or limitations of assays and methods.

Individual characteristics can have a significant impact on the utility of RIs. Many analytes measured in clinical chemistry can vary based on age, ethnicity, and sex. Using biological sex as an example, there are physiological differences between biological males and biological females that can lead to changes in laboratory measurements. Sex hormones, such as testosterone and oestrogen, vary based on biological sex and age. However, they also influence other laboratory analytes such as creatinine, haemoglobin, and cholesterol [43]. Examples of other analytes which are affected by these individual characteristics include calcium, phosphate, albumin, total protein, globulins, creatinine, urea, urate, sex hormone binding globulins, ferritin, and vitamin B12 [15, 44, 45]. However, this list is not exhaustive and there are many more examples in the literature.

Pregnancy can alter the concentrations of many analytes in blood. Sex hormones vary significantly throughout pregnancy and influence other physiological processes [46]. In early pregnancy, vasodilation of peripheral and renal vasculature leads to sodium and water retention which consequently increases plasma volume and causes haemodilution [47–50]. Concomitantly, the plasma osmostat is also altered meaning there is increased water retention. The result is that several analytes such as haemoglobin, haematocrit, and albumin are lower in pregnancy [46, 51].

If correctly identified, RIs can be partitioned by individual characteristics to provide more accurate information to clinicians, allowing them to make more informed decisions. However, there are many analytes affected by individual characteristics for which partitioned RIs do not yet exist. Rappoport *et al* compared clinical laboratory results across self-identified races and ethnicities (SIREs) for 50 of their most requested tests [52]. They identified differences in measurements across the SIREs for over half of these tests, however only one of these already had ethnicity specific RIs. Returning to the example above, partitioned RIs exist for sex hormones as would be expected given the obvious physiological differences [43]. However, reported RIs for albumin are not commonly partitioned by sex or age despite the known physiological impact of sex hormones on albumin concentration [5, 43, 53]. Similarly, trimester specific RIs now exist for some analytes such as thyroid hormones [40, 54, 55]. However, they do not exist for all analytes that are affected during pregnancy. A recent study in Taiwanese pregnant women identified alterations in blood levels of thyroid hormones, sex hormones, full blood count parameters, liver function tests, and renal function tests across trimesters yet specific RIs are not in routine use and have not been published in this population [56]. A major limitation faced by laboratory staff is that accounting for these characteristics during RI development can be very difficult as the number of subjects required will increase by 120 with each partition. This means it may not be possible for some laboratory services to offer partitioned RIs for some methods, unless they are able to verify RIs as described above, or use a different method to establish them as described in below.

The consequence of the difficulty in establishing partitioned RIs is that some clinical decisions could be made based on inaccurate RIs meaning the possibility of unnecessary interventions is increased. For example, literature often uses creatinine as an example of an analyte affected by individual characteristics [52, 57]. Due to this, it was previously recommended that the calculation for estimated glomerular filtration rate (eGFR) involved correction for race (African-American or European) [58]. However, evidence suggested that this correction factor was leading to over estimation of the GFR in some populations [59, 60]. This was possibly due to population differences when compared to the original measured population of African Americans. The consequence of this is that patients could be misclassified as having an eGFR within the RI when they actually have chronic kidney disease and are at a high risk of adverse outcomes [60]. Considering this evidence, new guidelines were published in 2021 recommending that ethnicity corrections were no longer used in the eGFR equation [61]. Clearly, partitioned RIs do have an important role to play, but only if they are established using strong data and are used appropriately.

On the other hand, there are some groups in which interpreting results using characteristic based RIs may be difficult. Tests such as haemoglobin, iron studies, troponin, and creatinine are affected by sex hormones or pubertal growth meaning partitioned reference ranges are used [62, 63]. Transgender individuals are those whose gender differs from that which was presumed for them at birth. It is estimated that around 0.6%–1.2% of the general population are gender diverse [64, 65]. Many of these individuals undergo gender-affirming hormone therapy which causes changes in regional body fat, lean body mass, libido, and body shape [66, 67]. Changes in muscle mass, fat mass, menstruation, and cell production can occur within 3 months of treatment initiation [66, 68–75]. Target RIs for sex hormones are those of the affirmed gender in individuals receiving hormone therapy. Of course, not all transgender individuals will be receiving hormone therapy, and those that are may be at different stages of their therapy. Adding to the complexity is the lack of detail in electronic patient records which can make this unclear for healthcare staff. Alternatively, some may ignore the RI and look at clinical outcome [70]. Unfortunately, others may just scan results and look for those highlighted for their attention meaning they may miss subtle details in these individuals. Evidence has shown that those receiving hormone therapy for gender affirmation experience changes in a range of tests such as the complete blood count, ferritin, renal function tests, prostate-specific antigen, and troponin that often fall close to the RI for the affirmed gender [70, 75–78]. Overall, this highlights the difficulty in using RIs to interpret results, especially if one does not understand or know the whole clinical situation. In 2013, the World Professional Association for Transgender Health recommended that electronic patient records and LIMS should include gender parameters such as presumed sex at birth, specific organs, and actual gender. Providing this information would avoid issues associated with misgendering and would allow the correct RI to be applied to the patient on the report [79]. However, this approach means there is reliance on the correct information being entered into the system and the wrong RI may still be applied if treatment isn’t considered. An alternative and possibly safer approach is to supply both male and female RIs so that the clinician can interpret results with all information available.

A common theme with the above limitations lies within the interpretation of RIs. The underlying issue is that they create an arbitrary dichotomous interpretation. In other words, interpretation of RIs allows for two options, within or outside of. Some may take this at face value to mean positive/negative, or diseased/non-diseased. Disease is a spectrum, and onset and progression are not sudden. Therefore, dichotomising an individual may not be applicable. Additionally, there are some situations where results may be within the RI when it is entirely inappropriate for the patient’s situation. For example, liver failure can cause reduced production of urea, whereas impaired renal function may reduce excretion of urea [80]. These disease states would cause reduced and elevated blood concentrations of urea respectively. Therefore, a urea result within the RI in a patient with known liver failure is something that requires further investigation due to the possibility of renal disease [1]. The best solution to this, and therefore many of the above issues, is proper training of all healthcare professionals involved in using RIs. Training that focuses on the limitations of RIs will allow individuals to interpret results in the context of the patient they are assessing, rather than the RI in isolation. Additionally, research into factors affecting results will enable a better understanding of the utility of RIs in specific situations or may even lead to generation of more RIs for different clinical scenarios.

## Alternatives to the Standard Reference Interval

An alternative to the standard method of establishing RIs described above is the indirect approach. This method uses patient data already collected and stored in a laboratory database to establish RIs. This process of using previously generated data is often referred to as data mining [81]. Indirect methods involve *a posteriori* study that excludes unhealthy subjects and establishes RIs using complex statistical algorithms [8]. These algorithms aim to identify a distribution amongst the data [81]. The indirect Hoffman method is an example of this. In short, a method is first used to detect and eliminate outliers. The cumulative frequency of each result is then determined, and a cumulative frequency graph is generated. Results from the linear portion of the graph are used to compute a regression line by linear regression. RIs are then determined from the linear regression equation [8]. The use of laboratory data raises concerns as most requests will be on individuals suspected to be in a “disease state” meaning data could be biased. However, this is dependent on the prevalence of the “disease state” in the population being studied, and this can be reduced in the extracted data through assessment of results from outpatients rather than inpatients that may suffer from more systemic disease or who may be receiving medication. Additionally, the results of other tests can be used as inclusion/exclusion criteria. In fact, it is argued that standard RIs derived from direct approaches may accidentally include those with subclinical disease, and the 1000 s of data points used in an indirect approach are statistically more significant than the 120 used in standard studies [8]. Indirect methods have been shown to have high comparability to peer reviewed RIs generated using the standard direct approach with no significant differences found in URLs and LRLs. For example, a study found that 18.6% of thyroid stimulating hormone results were above the URL whereas 5.9% were below the LRL [8]. This showed great similarity to the measured prevalence of subclinical hypothyroidism and subclinical hyperthyroidism which are 17% and 6% respectively [82].

The indirect method is a suitable alternative for laboratories to establish RIs as it has the advantage of not requiring subject recruitment and the design of a large study. This, in contrast to the direct approach of recruiting patients, means the cost is therefore far less for an indirect approach. It is also faster, as the data is already collected so should only require extraction and analysis, although one could argue that it has taken a long time to acquire the data. Additionally, the large amount of data allows for partitioning of RIs, which should allow for better outcomes for the patient. Arguably the most important benefit of the indirect approach is that the established RI should be derived from data that is both specific to a laboratory’s method and the population that they serve [81]. Critically, there should not be changes in either the population or method during the period of data collection. To verify RIs developed using the indirect approach, it is recommended that laboratories either measure samples from 20 individuals of known disease state or monitor the percentage of abnormal results and compare [81, 83].

As discussed above, RIs create a dichotomous interpretation based on data from a reference population. However, results may only be one significant figure apart and lead to them falling on opposite sides of either the LRL or URL. Therefore, a question that may be asked is whether the change in results is clinically significant? The result could be due to changes in the individual’s health, but it may also be due to pre-analytical factors or analytical variation, also known as analytical imprecision [84]. Biological variation is the concept that individuals have biological setpoints at around which biomarkers vary due to factors such as genetics, diet, age, and physical activity [85, 86] It is usually expressed as a coefficient of variation (CV), which is the standard deviation of a set of results divided by its mean, expressed as a percentage. This random variation can be due to intraindividual or within-subject variation (CV_i_) and interindividual or between-subject variation (CV_g_). Intraindividual variation is the physiological variation within a subject. In other words, it is the variation around their own biological setpoint. Interindividual variation is the variation between subjects. Therefore, it is the variation in biological setpoints across individuals [84, 87]. Biological variation is calculated by measuring analytes in a reference population that is in a steady state across a time period [87]. Estimations of the intraindividual and interindividual variation from studies are available on the European Federation of Clinical Chemistry and Laboratory Medicine website [88]. This information can be used by clinicians to determine if changes in results are clinically relevant or simply due to analytical variation. One method is to examine the reference change value (RCV) which is calculated as follows [84]:

$$RCV=2^{\frac{1}{2}}\times Z\times {\left({{CV}_{A}}^{2}+{{CV}_{I}}^{2}\right)}^{\frac{1}{2}}$$

Where Z is a multiplication factor related to the standard deviation, CV_a_ is the analytical imprecision, and CV_i_ is the intraindividual variation.

If the difference between two serial results in an individual is less than the RCV, then the variation is likely to be related to biological and analytical variations and is therefore not considered clinically significant. The RCV should be relatively straightforward to calculate using published CV_i_ values and a CV_a_ calculated using uncertainty of measurement data from the laboratory performing the test. A recent article from McCormack and Holmes summarises the use of RCVs and concludes that clinicians need to understand the concept of biological and analytical variation to know the meaning of results [89]. This method, in combination with RIs, has the potential to aide interpretation of laboratory results by clinicians.

The index of individuality is a ratio of the combined intraindividual variation and analytical imprecision to the inter-individual variation. In theory, it allows one to determine if RIs are sensitive enough to identify if a change in results for an individual patient is significant. If the index is >1.4, it suggests that RIs are appropriate for use in interpreting results. If the index is <0.6, it suggests that examining the difference between results may be more appropriate [87]. Essentially, it is suggesting that if the combined analytical imprecision and intraindividual variation are high compared to the interindividual variation, then using a RI is appropriate. If the interindividual variation is very high, then examining the change in results for an individual is more appropriate. This tool is less likely to be used for all results and could be more beneficial on a case-by-case basis. However, the RCV is arguably a more useful tool as it can determine if a result change is significant using less data than the index of individuality. By quoting intra- and inter-individual biological variation, laboratories can give clinicians more information than a RI alone to aid patient care.

Clinical decision limits (CDLs) are an alternative to RIs. CDLs are limits or cut-off values that are determined by clinical studies to be associated with a specific clinical risk or outcome [83]. Therapeutic intervals are a type of CDL that define the optimal range for therapeutic drugs [14]. CDLs are usually one value and are seen as a threshold with different patient pathways on either side. They are commonly established via clinical outcome studies and are communicated via published guidelines [14, 83]. The choice of a CDL is determined by the sensitivity and specificity of a test at that level. The sensitivity of a test is the probability of a positive test in an individual with the tested disease. It is calculated as the number of true positives divided by the number of true positives and false negatives combined. The specificity of a test is the probability of a negative test in an individual without the disease being tested for. It is calculated as the number of true negatives divided by the number of true negatives and false positives combined [90]. These parameters can be used to generate a receiver operator curve (ROC). In a ROC, sensitivity is plotted on the Y-axis and 1- specificity is plotted on the X-axis. Each point on the curve corresponds to a CDL. Therefore, the most appropriate CDL can be selected by balancing the sensitivity and specificity of a test. While RIs allow comparison to a reference population, CDLs are related to clinical outcomes. Therefore, they are more appropriate in some situations. For example, glycated haemoglobin, also known as HbA1c, is used to monitor glycaemia, response to treatment, and identify risk of complications in diabetes mellitus [91]. The RI for HbA1c is 20–42 mmol/mol however this is only reflects the measured concentrations in a reference population [92]. Clinical decision limits have been identified based on the risk for diabetes. Results <40 mmol/mol, 40–46 mmol/mol, and >46 mmol/mol reflect a low risk, an increased risk, and the presence of diabetes respectively. Additionally, targets of 53 mmol/mol and 64 mmol/mol exist for target treatment and limit change therapy respectively in individuals with diabetes that are being monitored [91]. The use of a RI in this situation would be inappropriate as results above the URL would not discriminate between an increased risk of diabetes or if an individual already has diabetes. RIs also wouldn’t be able to identify if treatment was successful in these individuals who may have persistently high levels [92]. Therefore, CDLs allow for different interventions to take place depending on the risk or outcome at certain levels and may be more useful than RIs for making decisions.

## Conclusion

In conclusion, RIs are a critical component of the clinical chemistry laboratory as they are required for result interpretation. Without them, clinicians would struggle to use results to aid patient care. However, there are significant limitations associated with their production and use. Training of healthcare staff is critical to raise awareness of these limitations to provide better patient care. However, the RI is undergoing a revolutionary period with the introduction of new methods for determination and the use of biological variation meaning healthcare staff will need to adapt. RIs will still play a massive role in the future of clinical chemistry, however incorporation of these factors means training will become even more vital.
